# Hyaluronic Acid Hydrogels Hybridized With Au-Triptolide Nanoparticles for Intraarticular Targeted Multi-Therapy of Rheumatoid Arthritis

**DOI:** 10.3389/fphar.2022.849101

**Published:** 2022-05-27

**Authors:** Chenxi Li, Rui Liu, Yurong Song, Youwen Chen, Dongjie Zhu, Liuchunyang Yu, Qingcai Huang, Zhengjia Zhang, Zeyu Xue, Zhenglai Hua, Cheng Lu, Aiping Lu, Yuanyan Liu

**Affiliations:** ^1^ School of Chinese Materia Medica, Beijing University of Chinese Medicine, Beijing, China; ^2^ Institute of Basic Research in Clinical Medicine, China Academy of Chinese Medical Sciences, Beijing, China; ^3^ School of Chinese Medicine, Hong Kong Baptist University, Hongkong, China

**Keywords:** rheumatoid arthritis, photothermal-chemo treatment, NIR imaging, mTOR/p70S6K pathway, TP-PLGA-Au@RGD/HA hydrogels

## Abstract

Rheumatoid arthritis (RA) is a chronic inflammatory disease, characterized by synovial inflammation in multiple joints. Triptolide (TP) is a disease-modifying anti-rheumatic drug (DMARD) highly effective in patients with RA and has anti-inflammatory properties. However, its clinical application has been limited owing to practical disadvantages. In the present study, hyaluronic acid (HA) hydrogel-loaded RGD-attached gold nanoparticles (AuNPs) containing TP were synthesized to alleviate the toxicity and increase therapeutic specificity. The hydrogels can be applied for targeted photothermal-chemo treatment and *in vivo* imaging of RA. Hydrogel systems with tyramine-modified HA (TA-HA) conjugates have been applied to artificial tissue models as surrogates of cartilage to investigate drug transport and release properties. After degradation of HA chains, heat was locally generated at the inflammation region site due to near-infrared resonance (NIR) irradiation of AuNPs, and TP was released from nanoparticles, delivering heat and drug to the inflamed joints simultaneously. RA can be penetrated with NIR light. Intraarticular administration of the hydrogels containing low dosage of TP with NIR irradiation improved the inflamed conditions in mice with collagen-induced arthritis (CIA). Additionally, *in vitro* experiments were applied to deeply verify the antirheumatic mechanisms of TP-PLGA-Au@RGD/HA hydrogels. TP-PLGA-Au@RGD/HA hydrogel treatment significantly reduced the migratory and invasive capacities of RA fibroblast-like synoviocytes (RA-FLS) *in vitro*, through the decrease of phosphorylation of mTOR and its substrates, p70S6K1, thus inhibiting the mTOR pathway.

## 1 Introduction

Rheumatoid arthritis (RA) is a systemic inflammatory disease characterized by persistent synovitis and aggressive joint destruction (Smolen et al., 2016). The conventional treatment of RA is the administration of disease-modifying anti-rheumatic drugs (DMARDs) aimed at improving inflammation and retarding disease progression (Ha et al., 2020). Triptolide (TP) as an epoxide diterpene lactone compound of DMARDs has been used clinically to inhibit the expression of adhesion molecules, metalloproteinases, and release of pro-inflammatory cytokine, thus demonstrating anti-inflammatory and cartilage protective effects in the treatment of RA (Li et al., 2015; Y.; Yang et al., 2016; Z. L.; Zhou et al., 2012). However, as a DMARD, its anti-arthritic effect has not been fully harnessed in clinical application, due to its multiple-organ toxicity, poor solubility, low stability, rapid excretion, and non-specific targeting (Cai et al., 2020; Li P et al., 2020). Therefore, it is imperative to develop novel targeted intraarticular delivery systems as an effective tool to specifically deliver TP within the inflammatory joint spaces with low dosage of drugs to reduce systematic side effects.

Combination of hydrogels and various near-infrared resonance (NIR) nanostructures with water-swollen and three-dimensional polymer networks has been increasingly used for targeted drug delivery, tissue regeneration, analgesia, chondroprotection, articular cartilage lubrication, and anti-inflammation effects (Agas et al., 2019; Burdick and Prestwich 2011; Mousavi Nejad et al., 2019). They can provide a favorable microenvironment for the growth, survival, and proliferation of cells owing to their numerous advantages including easy modification and less invasiveness during delivery (Vignesh et al., 2018; Li P et al., 2020). Drugs loaded in hydrogels or nanoparticles encapsulating drug molecules, growth factors, or any bioactive compounds are released in a sustained manner (Arun Kumar et al., 2015). Meanwhile, these drug-loaded hydrogels have structural and mechanical properties similar to several tissues and can potentially mimic the native extracellular matrix (ECM) structure especially (Vignesh et al., 2018; Vaca-Gonzalez et al., 2020). As an important component of ECM, hyaluronic acid (HA) is extensively used in the clinical treatment of RA owing to its high biodegradability, biocompatibility, and non-immunogenicity properties (Li C et al., 2020). It is involved in joint lubrication, cushions the load transmission across articular surfaces, provides a renewed source of HA for joint tissues and imparts antinociceptive and exhibits anti-inflammatory properties for synovial fluid (Concoff et al., 2017; Faust et al., 2018; Silva et al., 2018; Li P et al., 2020). HA can bind to CD44, which is over-expressed on the surface of synovial lymphocytes, fibroblasts, and macrophages in inflamed joints in RA. These cells promote RA progression by facilitating the migration of inflammatory cells and inducing activation of various effector cells (Naor and Nedvetzki 2003; Nedvetzki et al., 1999; Shen et al., 2015; Wang and Sun 201 7).

HA-based scaffolds, mechanically soft/tough hydrogels, *in-situ* hydrogels, and viscoelastic solutions have been successfully prepared through physical crosslinking or chemical modifications (Vignesh et al., 2018; Li P et al., 2020). However, the large pore sizes and high-water saturability of most hydrogels may cause a rapid and uncontrolled release of small drug molecules. Particularly, poorly water-soluble hydrophobic drugs tend to precipitate or be simply released from hydrogels in a rapid burst (Racine et al., 2017). To overcome these limitations, nanoparticles-based drug delivery systems have been entrapped within hydrogels to form composite hydrogels networks (Zhao et al., 2015). Metallic nanoparticles hybrid cross-linked hydrogels with superiorities of flexible fabrication, higher drug loading capacity, and sustained release may provide targeted delivery and controlled release for the treatment of RA (H. Lee et al., 2014; Racine et al., 2017). Notably, gold (Au) nanostructures have good stability, large loading surface-area-to-volume ratio, are easily synthesized, and have high surface functionalization, excellent optical and electrical properties, and remarkable light-heat transition ability (Hien et al., 2012; Van Phu et al., 2012). Among diversified Au nanostructures, nanoparticles, nano-shell, nanocages, and nanorods have attracted increased interest in various fields, including use as bio-sensors, drug delivery systems, imaging, tissue engineering, and photothermal therapy (Rao et al., 2018; Shakeri-Zadeh et al., 2021). In addition, Au nanostructures exhibit unique surface plasmon resonance and intense absorption in the NIR region, thus they can be used for *in vivo* imaging and photothermal therapy (W. Wang et al., 2020). NIR light enables the remote activation of the release process, and the drug release can be controlled by spatial or temporal control to occur at the desired time by illumination (Ji et al., 2013). Through the appropriate design of network structure and nanoparticles carriers, drug delivery systems can be engineered to provide a tailored drug release rate with sustained delivery.

For the treatment of RA, we developed TP-PLGA-Au@RGD/HA hydrogels directly intraarticularly administered into collagen-induced arthritis (CIA) mice to explore whether drug accumulation occurs at the inflamed area, which may potentially ensure a higher therapeutic concentration, minimize side effects, and enhance anti-inflammatory effects. After the degradation of HA chains, TP-PLGA-Au@RGD nanoparticles (TP-PLGA-Au@RGD NPs) were exposed to the surrounding environment. RGD peptides as a cell adhesion sequence found in the ECM are easily recognized by integrin overexpressed cells (Sheikh et al., 2021; M.; Yang et al., 2021). Eight integrins recognize RGD sequences in natural ligands, such as α5β1 and αvβ3 integrins (Sheikh et al., 2021). Due to RGD peptides conjugated to the TP-PLGA-Au@RGD NPs, which bind α_v_β_3_ integrins expressed on angiogenic vascular endothelial cells at sites of inflammation to induce targeted delivery property (Hynes 2002). Upon NIR exposure, the presence of AuNPs resulted in heat generation; thus; drugs were released from nanoparticles. This effect resulted in photothermally controlled drug delivery and release. Compared to conventional treatment, the application of synthesized sustained-release hydrogels earned superior therapeutic effects with low dosage of TP in CIA mice.

Furthermore, *in vitro* experiments were applied to deeply verify the antirheumatic mechanisms of TP-PLGA-Au@RGD/HA hydrogels. These synthesized hydrogels were specifically targeted to inflamed joints, and exert superior anti-arthritic effects on reducing destructive joint inflammation mediated by RA fibroblast-like synoviocytes (RA-FLS), which were applied for in-depth signaling pathway investigation used to reveal the mechanism of targeted drug delivery in inflamed joints, owing to their characteristic pathogenic behavior, such as aggressive phenotype, mediation of inflammation and destruction of the joint (Dai et al., 2018). The mammalian target of the rapamycin (mTOR) signaling pathway plays an important role in regulating cell growth, proliferation, differentiation, and inflammation in many pathophysiological conditions (Dai et al., 2018; Chen et al., 2021). Moreover, the mTOR signaling pathway is involved in the regulation of FLS invasion, which shows aggressive characteristics similar to tumor cells (Firestein 1996). Therefore, the anti-inflammatory effects of TP-PLGA-Au@RGD/HA hydrogels on RA-FLS may occur via the regulation of the mTOR/phosphorylation p70S6 kinase (mTOR/p70S6K) signal pathway, which maintains in the abnormal activated state, resulting in high expression of anti-apoptosis genes and the subsequent impact on multiple downstream effector molecules (Wu et al., 2017; Feng and Qiu 2018). The present study, therefore, aimed to investigate the anti-inflammatory effects of TP-PLGA-Au@RGD/HA hydrogels in CIA mice and to reveal the potential signaling pathways involved.

## 2 Materials and Methods

### 2.1 Materials

HA was obtained from Aladdin. SH-PEG, Tyramine hydrochloride, Sulfo-NHS, branched polyethyleneimine (PEI, average Mw = 10,000) and TP was supplied by Shanghai Yuanye Bio-Technology Co., Ltd. Pluronic F-127 was supplied by Beyotime Biotechnology Co., Ltd. Horseradish Peroxidase (HRP) and bovine testicular hyaluronidase were purchased from the Solarbio Science & Technology Co., Ltd. (Beijing). RGD peptide was obtained from Xi’an Biological Technology Co., Ltd. Poly (DL-lactic-co-glycolic acid) (PLGA) and 1-ethyl-3-(3-dimethylaminopropyl) carbodiimide (EDC) were supplied by Shanghai Macklin Biochemical Technology Co., Ltd.

### 2.2 Synthesis of TP-PLGA-Au@RGD NPs and Tyramine-Modified HA

6 mg of TP and 100 mg of PLGA were dissolved in 20 ml of dichloromethane under magnetic stirring. 200 ml of distilled water containing Pluronic F-127 (200 mg) was slowly added drop-wise to serve as a stabilizer of the mixture. The mixture was emulsified by ultrasonication after 1 h to ensure thorough mixing of the oil and water phase, followed by evaporation of dichloromethane under stirring for 2 days. TP-Poly (DL-lactic-co-glycolic acid) nanoparticles (TP-PLGA NPs) were obtained by centrifugation. TP-PLGA NPs were added to PEI aqueous solution (1 mg ml^−1^) and stirred for 30 min then centrifuged. After centrifugation, 50 ml AuNPs solution was added to the TP-PLGA-PEI NPs solution and subjected to vigorous stirring for 20 h. TP-PLGA-Au NPs were then obtained through centrifugation. 5 nm Au/Citrate dissolved in water at a concentration of 1 mg/ml was supplied by Deke Technology Co., Ltd. (Beijing, China). TP-PLGA-Au NPs was released from the substrate into 1wt% SH-PEG-COOH solution through sonication and collected by centrifugation at 10,000 rpm. The carboxylic-acid-terminated TP-PLGA-Au@RGD NPs, EDC (8 mg)/NHS (8 mg), and RGD (6 mg) were dissolved in 18 ml of PBS (pH 7.4) and stirred at room temperature. The reaction mixture was then left at room temperature for the RGD peptides to covalently bind to the -COOH group of SH-PEG-COOH chains adsorbed to AuNPs. TP-PLGA-Au@RGD NPs were obtained by centrifugation after 24 h and the supernatant containing unreacted RGD was discarded.

The process for the synthesis of TA-HA is presented in Figure 3A. 10 mg of HA was dissolved in 2 ml of deionized water. Then, 64.5 mg of tyramine hydrochloride, 9.6 mg of EDC, and 10.9 mg of NHS were added to the HA solution. The pH of the mixture was adjusted to 4.7 using 1 M HCl or 1 M NaOH and then stirred for 24 h. Then adjusted the pH to 7.0 using 1 M HCl or 1 M NaOH. The mixture solution was transferred into a dialysis bag (3.5 k Da) for 3 days to ensure the unreacted tyramine hydrochloride and other salts were completely removed. The obtained TA-HA was freeze-dried.

### 2.3 Preparation of TP-PLGA-Au@RGD/HA Hydrogels

Preparation of the hydrogels was conducted as described previously (Bian et al., 2016; Kheirabadi et al., 2015; M. Kurisawa, J. Chung, Y. Yang, S. Gao, and H. Uyama, 2005). The TA-HA (17 mg) was dissolved in 2 ml of PBS (pH = 7.4) at room temperature. The TA-HA was allowed to completely dissolve in PBS, then 1 mg of TP-PLGA-Au@RGD NPs was gently added to the prepared solutions and the mixture was ultrasonicated for 10 min. Furthermore, HRP was dissolved in PBS (10 μL, 0.2 mg/ml) and then added to the mixture of TA-HA and TP-PLGA-Au@RGD NPs. Hydrogen peroxide (H_2_O_2_) was then added to the obtained mixture. The mixture was stirred gently, resulting in the formation of TP-PLGA-Au@RGD/HA hydrogels.

### 2.4 Characterization of the Materials

The TA-HA was characterized using an ^1^HNMR spectrometer (Agilent-600M). A Fourier transform infrared spectrometer (FT-IR, Thermo Fisher Scientific, United States) was used to record the infrared spectrum. Zeta-potential and size were determined by dynamic light scattering at 25°C to explore the characteristics of the synthesized TP-PLGA-Au@RGD NPs. Morphology of the TP-PLGA-Au@RGD NPs and TP-PLGA-Au@RGD/HA hydrogels was explored using a transmission electron microscope (TEM, HT7700) and a scanning electron microscope (SEM, Apreo2). A UV–vis–NIR spectrometer was used to determine the UV–vis–NIR absorption spectrum of the AuNPs and TP-PLGA-Au@RGD NPs.

### 2.5 Photothermal Property and *in vitro* Release

TP-PLGA-Au@RGD/HA hydrogels containing TP-PLGA-Au@RGD NPs (1 mg) in a centrifuge tube were under 808 nm NIR irradiation for 10 min. The cooling curve was got after the laser was turned off.

The encapsulation efficiency parameter of TP-PLGA-Au@RGD NPs refers to the percentage of drug entrapped with respect to the total amount of drug added through the nanoparticle preparation process. The quantification was performed by UV spectrophotometry at a wavenumber of 220 nm (*n = 5*). The TP content inside the nanoparticles was expressed as the ratio of the amount of TP found in the nanoparticles and the total initial amount of TP used in the preparation of the batch and was represented as a percentage, according to the following equation (Mousavi Nejad et al., 2019):

$$\text{ Encapsulation efficiency}=\frac{\text{Amount of TP in nanoparticles}}{\text{Total initial amount of TP}}\times 100\text{\%}\text{.}$$

1 mg of TP-PLGA-Au@RGD NPs was added to 5 ml of PBS solution containing 5 unit/mL hyaluronidase and allowed to fully disperse. The mixture was then transferred into a dialysis bag (3.5 k Da). The dialysis bag was immersed in a small glass tube containing 20 ml of PBS and was slightly shaken constantly at 150 rpm. NIR irradiation for 10 min performed before shaking. Under the same conditions, TP-PLGA-Au@RGD/HA hydrogels containing 5 unit/mL hyaluronidase and TP-PLGA-Au@RGD NPs (1 mg) were loaded into a dialysis bag (3.5 k Da). The dialysis bag was placed in a tube containing 20 ml of PBS solution. 3 ml of fresh PBS solution was added to replace the release medium at determined intervals at 37 C to maintain sustained-release conditions and NIR irradiation for 10 min was performed before shaking. UV–vis spectrophotometry was used to determine the amount of released drug at a wavelength of 220 nm.

TP-PLGA-Au@RGD/HA hydrogels containing 5 unit/mL hyaluronidase and TP-PLGA-Au@RGD NPs (1 mg) were loaded into a dialysis bag (3.5 k Da). The dialysis bag was placed in a tube containing 20 ml of PBS solution. 3 ml of fresh PBS solution was added to replace the release medium at determined intervals at 37 C to maintain sustained-release conditions, and NIR irradiation and non-NIR irradiation for 10 min were performed before shaking.

### 2.6 Cell Studies

#### 2.6.1 Preparation and Culture of RA-FLS

RA-FLS and growth medium were purchased from Cell Applications (Beijing Longyue Biological Technology Development Co., Ltd.). RA-FLS obtained from passages 5 to 9 were seeded onto 96-well plates at a density of 1 × 10 ^4^ cells/mL. Cells were cultured in Dulbecco’s Modified Eagle Medium (DMEM) supplemented with 4.5 g/L glucose, 100 μg/ml streptomycin, 100 IU/ml penicillin, and 10% fetal bovine serum. Cells were cultured in a humidified 37 C incubator with oxygen and 5% CO_2_.

#### 2.6.2 CCK-8 Assay

Cells were cultured in DMEM supplemented with 4.5 g/L glucose, 100 IU/ml penicillin, 10% fetal bovine serum and 100 μg/ml streptomycin. RA-FLS were collected and seeded onto 96-well plates at a density of 5 × 10^3^ cells. A CCK-8 assay was performed to determine the effect of freeze-dried hydrogels on the proliferation of RA-FLS. All samples were diluted with the corresponding culture medium. Cells were treated with different concentrations of drug and for different durations (24 h, 48 h). After treatment, 10 μL CCK-8 solution (Shanghai Yuanye Bio-Technology Co., Ltd.) was added to each well and incubated at 37 C, at 5% CO_2_ for 1 h. The absorbance was determined at 450 nm using an enzyme-linked immunosorbent assay reader (BioTek Instruments, Inc., Winooski, VT, United States).

#### 2.6.3 Western Blot Analysis

The freeze-dried TP-PLGA-Au@RGD/HA hydrogels were diluted with the corresponding culture medium for cell studies. PExpression of mTOR, p70S6K, p-mTOR and p-p70S6K proteins was determined by western blotting (Abcam, United Kingdom). Total protein was extracted from cells using Cell Lysis Buffer (Abcam, United Kingdom). Western protein marker was purchased from HaiGene. 30 μg of the total protein sample was loaded and separated on a 4–20% SDS–PAGE gel under reducing conditions. Samples were then transferred onto nitrocellulose membranes, and the membrane was blocked with 5% skim milk. Nitrocellulose membranes were incubated with primary antibodies and secondary antibodies. Western blots were analyzed using chemiluminescence. Band intensity on the western blot was quantified by densitometric scanning using ImageJ software (National Institutes of Health).

### 2.7 Animal Studies

#### 2.7.1 Induction of CIA Mice

The animal experiments were carried out under the Guidelines of Ethical and Regulatory for Animal Experiments formulated by the Institute of Basic Theory, China Academy of Chinese Medical Sciences (License Number: SCXK (Beijing) 2016-0011, SYXK (Beijing) 2017-0033). Bovine type II collagen (200 μg, Sigma-Aldrich, Shanghai) emulsified in complete Freund’s adjuvant (200 μg, Sigma-Aldrich, Shanghai) was intradermally injected into male DBA/1J mice (8 weeks old, Medcona) to induce rheumatoid arthritis. Furthermore, 100 μg bovine type II collagen was added to incomplete Freund’s and administered to mice on day 21 after the primary immunization, to boost the intradermal injection.

#### 2.7.2 Treatment of CIA Mice

After successfully establishing the rheumatoid arthritis model, saline (G1) and 10 mg/kg of TP solution (G2) were intraarticularly administered to CIA mice. Moreover, TP-PLGA-Au@RGD/HA hydrogels (G3 and 4) containing 10 mg/kg of TP-PLGA-Au@RGD NPs were intraarticularly administered to mice. After administration of TP-PLGA-Au@RGD/HA hydrogels, G4-treatment combined with 808 nm NIR irradiation was conducted for 10 min (*n = 6 mice in each group*). The arthritis index is the sum of scores for four-paw scores and the highest score is 16 points (M. Lee et al., 2012). The condition of evaluated paws was determined with scores from 0 to 4 using the following scale: 0 = no erythema and swelling, 1 = erythema and mild swelling, 2 = erythema and mild swelling extending from the tarsals to the ankle, 3 = erythema and moderate swelling extending from the metatarsal to ankle joints, and 4 = erythema and severe swelling of the digits, foot and ankle or ankylosis of the limb. Mice were monitored twice a week for 4 weeks after administration.

### 2.7.3 *In Vivo* NIR Imaging

The prepared TP-PLGA-Au@RGD NPs solution (at a concentration of 2 mg/ml in 2 ml of deionized water) was magnetically stirred continuously for 12 h at room temperature, and then, 50 μL of DMSO solution containing 10% (w/w) cyanine7 (Cy7) was added to the solution. The resulting mixture was then centrifuged at 4 C (12,000 rpm for 10 min). TP-PLGA-Au@RGD-Cy7/HA hydrogels and Cy7 were intraarticularly administered to CIA mice. An IVIS Spectrum (Carestream Health Fx Pro/FX) *in vivo* fluorescence imaging system was used to observe the mice.

### 2.7.4 Histological Analysis

Mice were sacrificed after 28 days of each treatment for histological analyses. The joints were harvested from the mice, and fixed in 10% buffered formalin saline at 4°C for a week. Decalcified joints were embedded in paraffin blocks and 4 μM-thick paraffin sections were obtained. Hematoxylin and eosin (H&E) were used to stain the joint tissues sections. Changes in synovial inflammation were scored on a scale of 0–4 (Camps et al., 2005). Deparaffinized sections were incubated with specific antibodies against IL-Iβ, IL-6, and TNF-α, followed by incubation with the appropriate peroxidase/DAB secondary antibodies (Abcam). Expression levels of different cytokines in the synovial tissues were semi-quantitatively on a 3-point scale (Lee et al., 2009). 0 = no expression, 1 = mild expression, 2 = moderate expression, and 3 = abundant expression of a cytokine. All histological analyses were performed independently and blindly by two evaluators. The average of the independent scores was then calculated.

### 2.7.5 Microcomputed Tomography

The paws of experimental mice in each group were scanned by using a micro-CT system (SkyScan 1176, Belgium). NFR Polarys software (Exxim Computing Corporation, Pleasanton, United States) was used to reconstruct and explored the three-dimensional structure of scanned paws. Images were acquired at 80 k Vp, 5 s/frame, and 150 mA, with 360 views. The three-dimensional bone volume (BV) including metatarsal bones and phalanges was determined using Aquarius software (version 4.4.6, TeraRecon, Inc.) to explore the volumetric change of arthritis joints.

#### 2.7.6 *In Vivo* Toxicity of Hydrogels

TP-PLGA-Au@RGD/HA hydrogels were administered intraarticularly into CIA mice (*n* = 6). Mice were then sacrificed after 28 days of each treatment, and the major organs (liver, heart, spleen, kidney, and lung) were harvested.

### 2.8 Statistical Analyses

Images and data were obtained from three independent experiments. Data were expressed as mean ± standard deviation. Statistical analyses were performed using GraphPad Prism 8.0. Differences among groups were determined by analysis of variance (ANOVA) and followed by Tukey’s post hoc test.

## 3 Results and Discussion

### 3.1 Characterization of TP-PLGA-Au@RGD/HA Hydrogels

**FIGURE 1:**
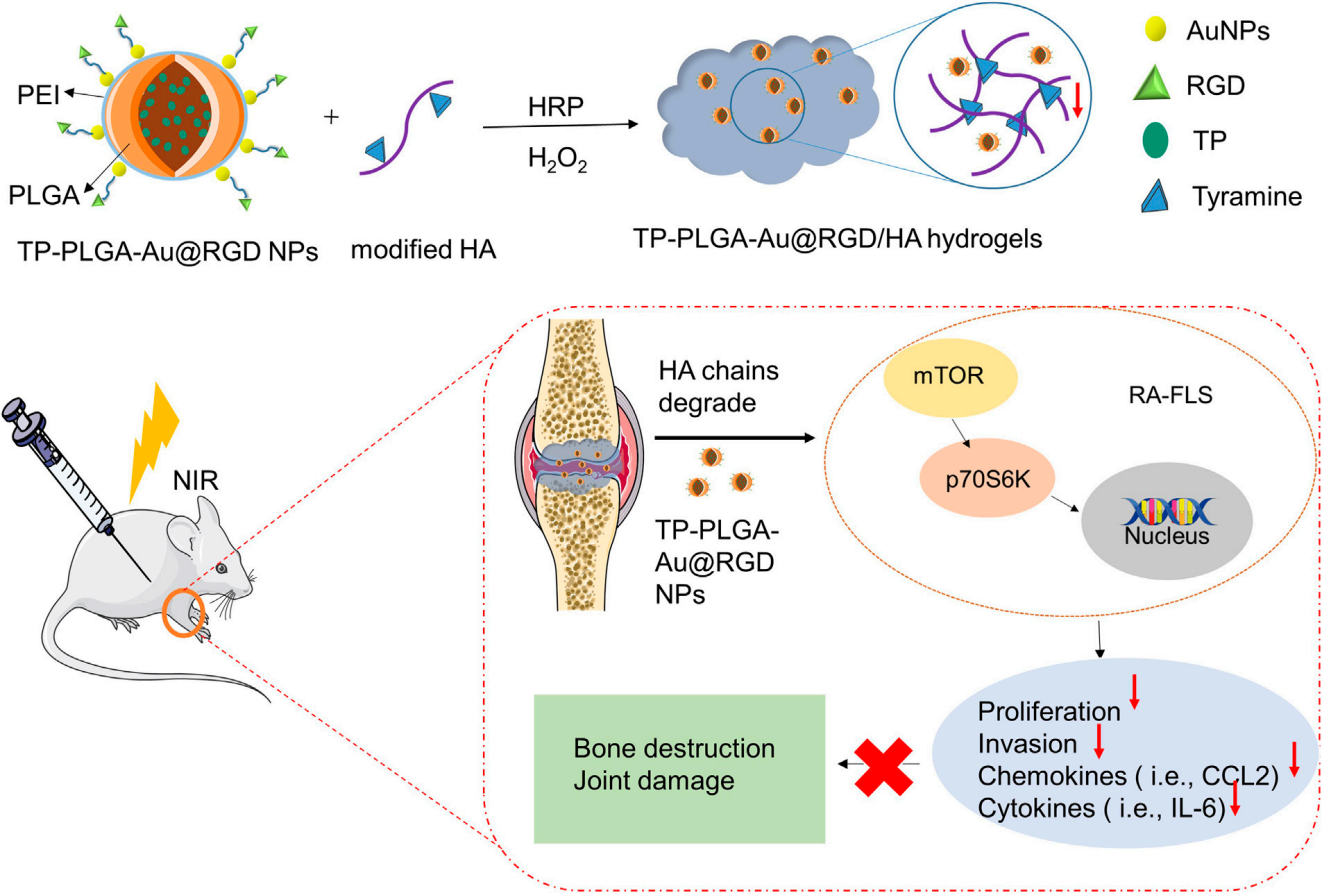
Preparation process of TP-PLGA-Au@RGD/HA hydrogels and schematic illustration of the anti-inflammatory effect of TP-PLGA-Au@RGD/HA hydrogels in CIA mice.

**FIGURE 2:**
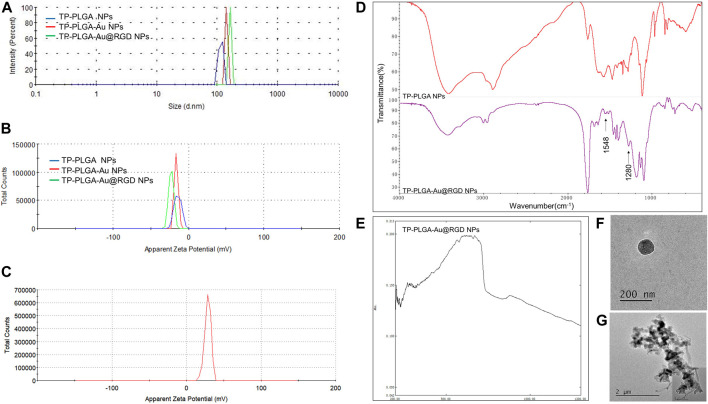
**(A)** Size distribution and **(B)** zeta potential of TP-PLGA NPs, TP-PLGA-Au NPs, and TP-PLGA-Au@RGD NPs. **(C)** Zeta potential of TP-PLGA-PEI NPs. **(D)** FT-IR spectra of the TP-PLGA NPs and TP-PLGA-Au@RGD NPs. **(E)** UV–vis–NIR spectra of TP-PLGA-Au@RGD NPs. **(F)** TEM image of TP-PLGA-Au@RGD NPs. **(G)** TEM image showed that TP-PLGA-Au@RGD NPs were embedded within the matrix of hydrogels.

A schematic representation for the preparation of hydrogels is presented in Figure 1. TP-loaded PLGA NPs absorb to PEI-linked AuNPs with RGD peptides at the terminals for targeted delivery. Then, the above synthesized TP-PLGA-Au@RGD NPs are embedded within the network of modified HA (TA-HA) hydrogels to form TP-PLGA-Au@RGD/HA hydrogels. The detailed preparation of TP-PLGA-Au@RGD NPs is as follows: TP-PLGA NPs were initially prepared. TP-PLGA NPs were negatively charged, with a zeta potential of −16.3 ± 1.1 mV. The negative charge of the nanoparticles implied that they can adsorb positively charged PEI for subsequent attachment of negatively charged AuNPs. The zeta potential for TP-PLGA-PEI NPs was 28.5 mV (Figure 2C). TP-PLGA-PEI NPs were modified with AuNPs to obtain TP-PLGA-Au NPs (Liu et al., 2016). TP-PLGA-Au NPs was released from the substrate into 1 wt% SH-PEG-COOH solution through sonication and collected by centrifugation. Then, the RGD peptides bind covalently to the -COOH group of the SH-PEG-COOH chains adsorbed to carboxylic-acid-terminated AuNPs, which binds αvβ3 integrins expressed on angiogenic vascular endothelial cells at sites of inflammatory to induce targeted delivery property (Hynes 2002). The FT-IR displayed the significant peaks of TP-PLGA NPs and TP-PLGA-Au@RGD NPs (Figure 2D), which showed 3415.74 and 1567 frequencies indicating the formation of the conjugate of the PLGA. Meanwhile, the bands at 1548 and 1280 cm^−1^ were attributed to the -C=O and -CO-NH_2_ groups for RGD. UV–vis–NIR absorbance spectra of AuNPs was around 516 nm and the absorbance of solid TP-PLGA-Au@RGD NPs ranged between 500 and 600 nm indicating the presence of AuNPs attached to the TP-PLGA-PEI NPs (Figure 2E and Supplementary Figure S2). TP-PLGA-Au@RGD NPs maintained their spherical shape (Figure 2F) and the average diameter of TP-PLGA-Au@RGD NPs increased to ∼164.2 nm and the zeta potential was −23 ± 1.8 mV (Figures 2A,B).

**FIGURE 3:**
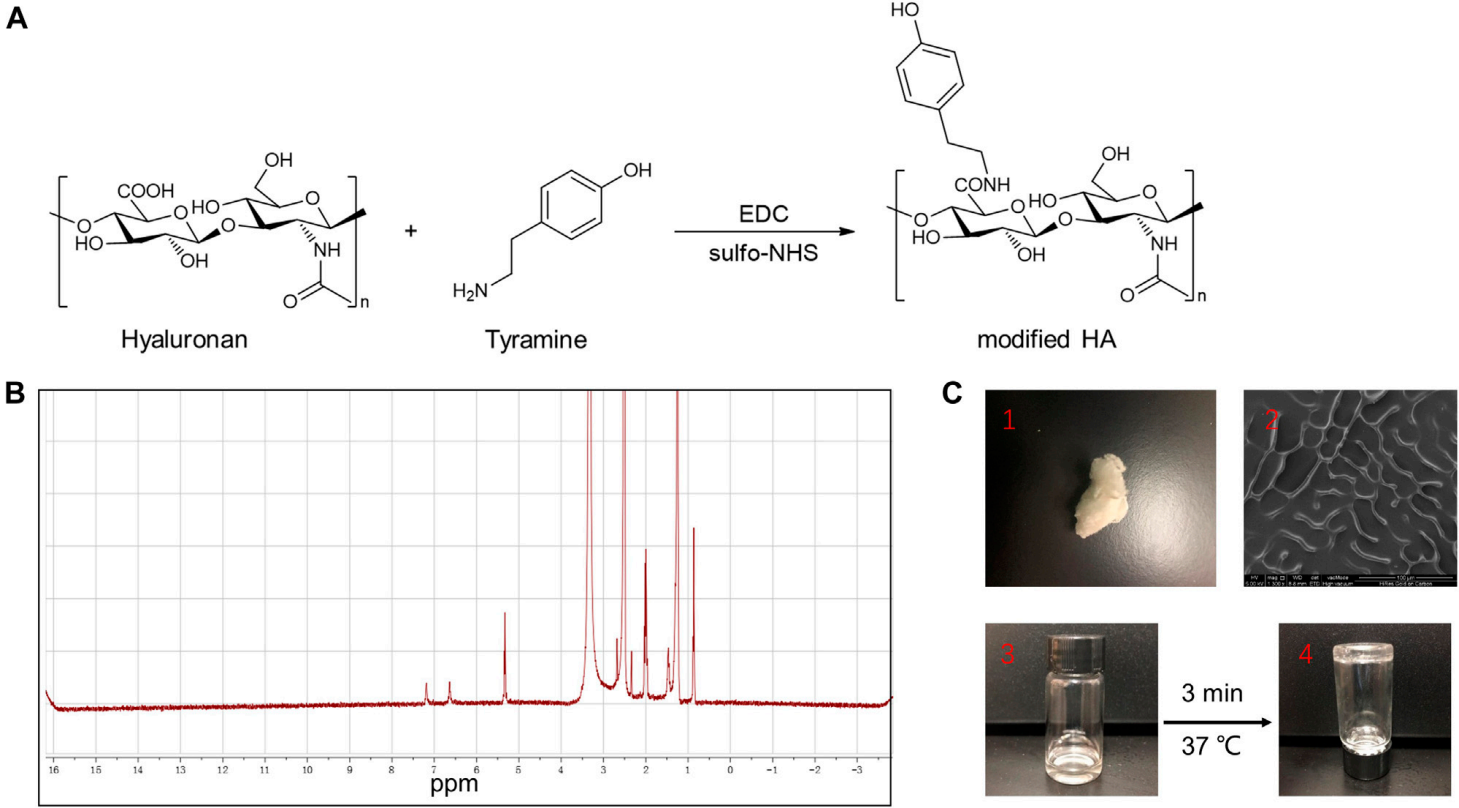
**(A)** Synthesis of TA-HA. **(B)** 1H NMR spectra of modified HA. (**C**-1) The morphology of the freeze-dried TA-HA, was white flocculent. (**C**-2) SEM image of TA-HA. (**C**-3) The morphology of freeze-dried TA-HA in PBS solution was transparent, colorless, and fluid. (**C**-4) Under the catalysis of HRP and H2O2, TA-HA formed hydrogels after 3 minutes, and the morphology of hydrogels had a transparent and non-fluid semi-solid nature.

TA-HA conjugate was synthesized and exploited to prepare TA-HA hydrogels for the applications in the treatment of RA. TA-HA conjugate was synthesized by amide bond formation between the amine group of tyramine and the carboxyl group of HA after activation with EDC and sulfo-NHS (M. Kurisawa et al., 2005), as shown in Figure 3A. ^1^H NMR spectra of the TA-HA were presented (Figure 3B and Supplementary Figure S2). Peaks observed at 6.69 and 7.2 ppm corresponding to the aromatic protons of TA being integrated. By calculating the integrated area ratio of the aromatic protons (*δ* = 6.69 and 7.2 ppm) and the HA methyl protons (*δ* = 1.9 ppm), the degree of substitution of TA was 13 (Number of TA per 100 repeating units of HA). SEM images showed the morphology of modified HA (Figure 3C2). The freeze-dried TA-HA conjugates were white flocculent (Figure 3C1). In Figure 3C3, the morphology of modified HA in PBS solution was transparent, colorless, and fluid. Under the catalysis of HRP and H_2_O_2_, TA-HA formed hydrogels after 3 min, and the morphology of hydrogels had a transparent and non-fluid semi-solid nature in Figure 3C4. In Figure 2G, the TEM image showed that synthetic nanoparticles were embedded in the hydrogels.

### 3.2 Drug Release and Photothermal Effects of Hydrogels

**FIGURE 4:**
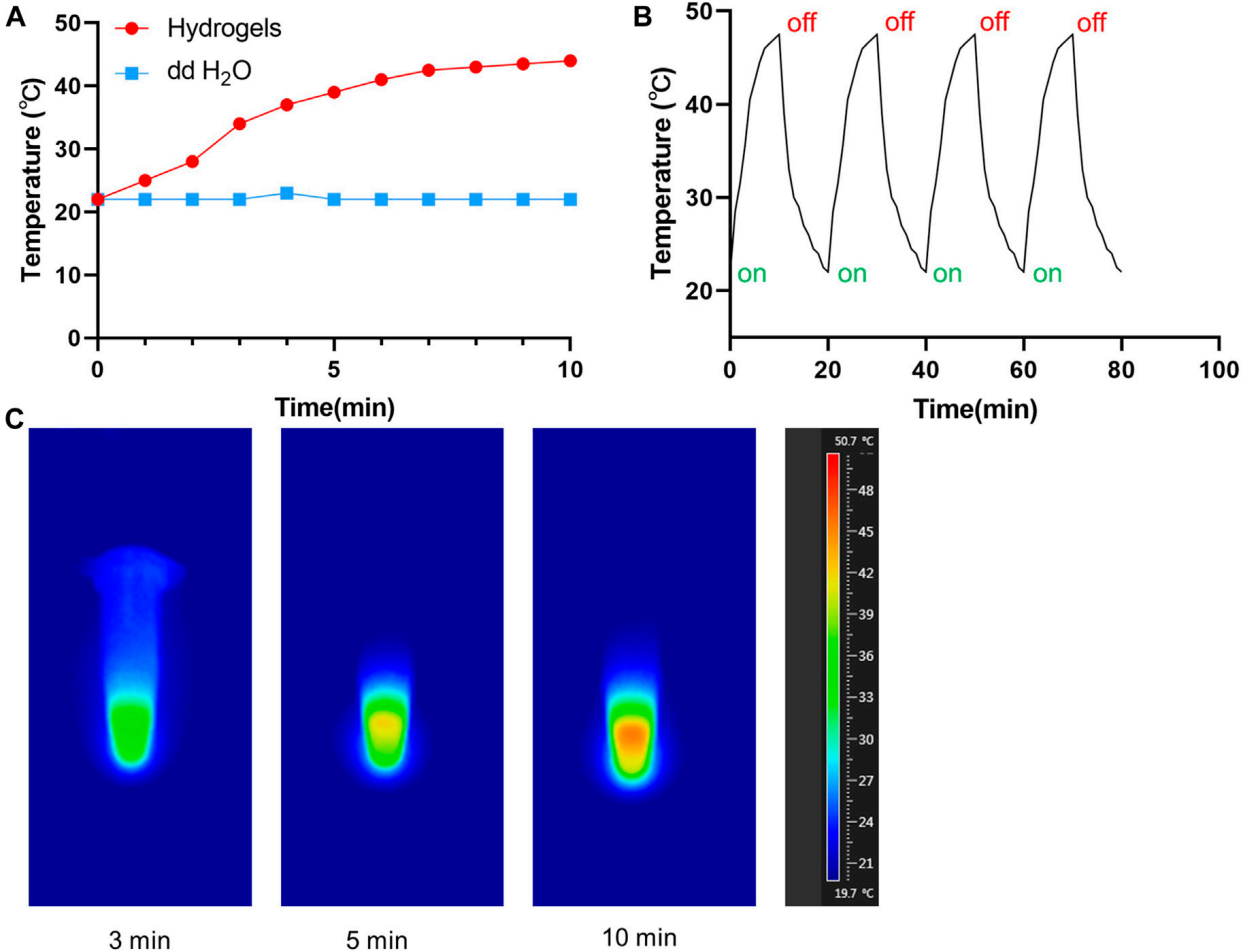
**(A)** Temperature curves of TP-PLGA-Au@RGD/HA hydrogels (808 nm). **(B)** Temperature evolution of TP-PLGA-Au@RGD/HA hydrogels over 4 on-off cycles with 808 nm laser irradiation. **(C)** Thermal images of TP-PLGA-Au@RGD/HA hydrogels under 808 nm laser irradiation at different time points.

In order to evaluate the photothermal effects of TP-PLGA-Au@RGD/HA hydrogels *in vitro*, used the 808 nm laser to irradiate the solution and monitoring the variation of temperature (Figure 4A). With the lasting of the irradiation time, the temperature gradually increased, and the temperature can reach 47.2°C after 10 min of irradiation. When the temperature tended to be constant, the hydrogels would not be irradiated. After 4 circulations of on-off, the maximum temperature of hydrogels can still maintain 47 C, indicating that hydrogels have good photothermal stability (Figure 4B). The infrared imaging device recorded the change of temperature change. As shown in Figure 4C, the temperature of the TP-PLGA-Au@RGD/HA hydrogels solution increased from 22 to 47°C after 10 min of irradiation.

**FIGURE 5:**
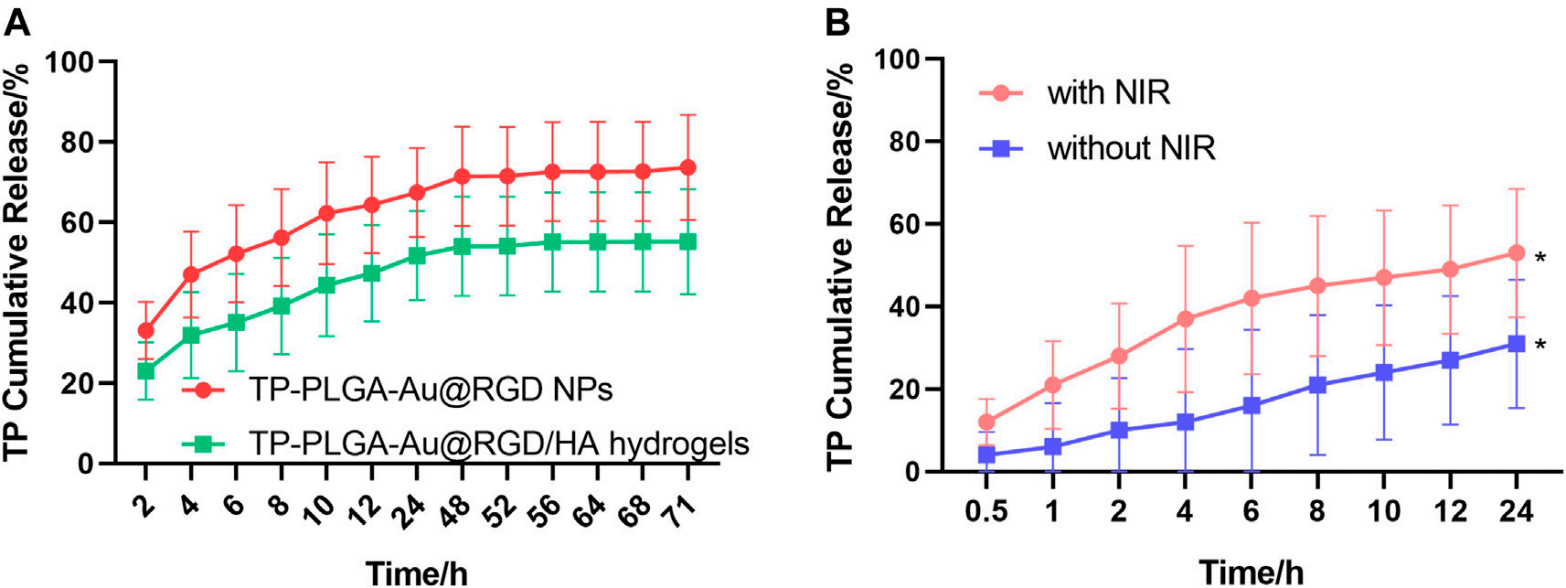
**(A)** Profiles of TP release from TP-PLGA-Au@RGD/HA hydrogels and TP-PLGA-Au@RGD NPs under initial NIR irradiation for 10 min. **(B)** Profiles of TP release from TP-PLGA-Au@RGD/HA hydrogels with or without NIR irradiation for 10 min. Data are expressed as mean values for *n* = 3, and error bars represent the standard deviation of the means (*p < 0.05).

The release behavior of TP-PLGA-Au@RGD/HA hydrogels and TP-PLGA-Au@RGD NPs was explored through *in vitro* release studies. The results indicated longer sustained delivery of drug from the hydrogels. The encapsulation efficiency of the prepared TP-PLGA-Au@RGD NPs was 30 ± 5%. Analysis of TP-PLGA-Au@RGD NPs showed that the release time of TP was 3 days, and the burst release rate was more than 60% within 12 h (Figure 5A). The release profile of the drug from TP-PLGA-Au@RGD/HA hydrogels showed that the sustained release time of TP was 3 days, and the burst release rate of the drug within 72 h was approximately 60%. This release behavior was attributed to the embedding of nanoparticles within the network of hydrogels. A relatively dense and stable hydrophilic shell was formed by the long HA chain with multiple interaction sites. The release of the drug from TP-PLGA-Au@RGD/HA hydrogels was slower, which may be attributed to the combined efficacy of TP-PLGA-Au@RGD NPs in the drug release and coacervate system. (Jin and Kim 2008). This finding indicated that the hydrogels can be used as a drug delivery system with long-term sustained release of the nanomedicine.

TP-PLGA-Au@RGD NPs were exposed to the surrounding environment after the degradation of HA chains. The release rate of TP was determined at 37 C to explore the efficacy of NIR irradiation and non-NIR irradiation (Figure 5B). The NIR light is in the range from 700 to 1000 nm, the long wavelength of NIR light penetrated deeper into human tissues and resulted in less detrimental to healthy cells with the reduced absorption and scattering of NIR in water and biological substances (Fedoryshin et al., 2014). AuNPs have been reported to absorb NIR light to transfer heat efficiently for photothermal therapy of cancers (Liang et al., 2016). Under the irradiation of NIR with 808 nm wavelength, the absorption due to the longitudinal surface plasmon resonance of loaded AuNPs generated heat and triggered the accelerated drug release from the nanoparticles, which exerted active and control of drug release via the non-contact mode (Zhang et al., 2017). The release profile of TP from TP-PGA-Au@RGD/HA hydrogels with non-NIR irradiation was linear, indicating that the release rate of TP was approximately constant. However, the release rate was increased and a burst release of TP was induced for 12 h after NIR irradiation for 10 min. These results indicated that NIR irradiation can control the TP release rate from TP-PLGA-Au@RGD/HA hydrogels.

### 3.3 Proliferation Inhibition Effect of Prepared Materials on RA-FLS *In Vitro*

**FIGURE 6:**
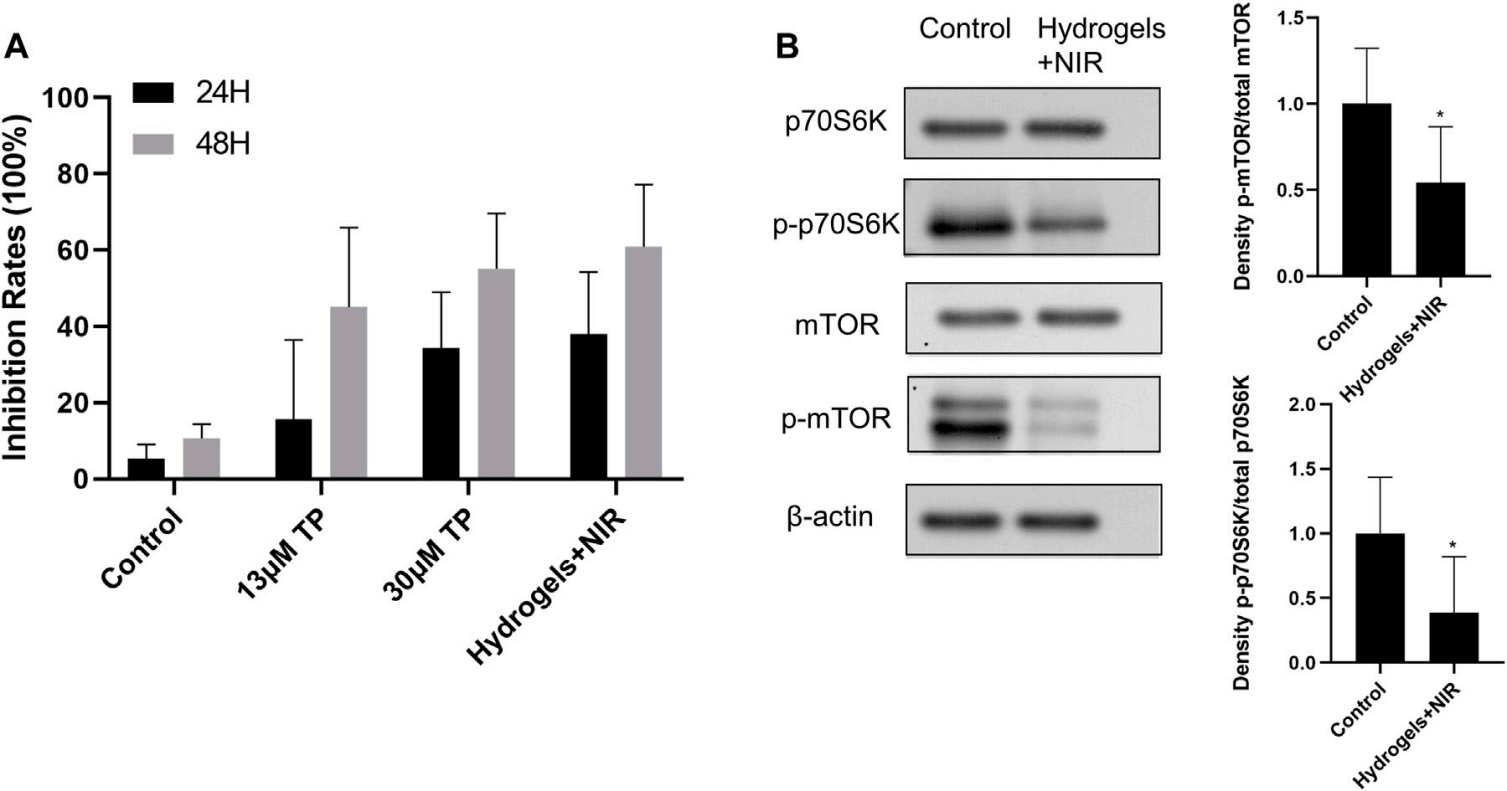
**(A)** Anti-proliferative effect of TP-PLGA-Au@RGD/HA hydrogels on RA-FLS. The data were obtained from three independent experiments and are expressed as mean ± SD. **(B)** TP-PLGA-Au@RGD/HA hydrogels reduced levels of phospho-mTOR (p-mTOR), with a p-mTOR/total median reduction of 54%, and levels of phosphorylation of mTOR targets with a phospho-p70S6K (p-p70S6K/total median reduction of 38%, indicating inhibition of the mTOR pathway. Data are expressed as mean ± SEM values. *P < 0.05 compared with the blank group.

RA-FLS were used to explore the anti-proliferative effects of TP-PLGA-Au@RGD/HA hydrogels combined with NIR irradiation. The *in vitro* cell proliferation experiment for RA-FLS was conducted for 48 h because the dissolution of the hydrogels reached a plateau of release after 48 h during *in vitro* drug release tests. CCK-8 assay results showed that proliferation of RA-FLS was significantly inhibited by hydrogels (Figure 6A). Cells were treated with 13 μM TP solutions, 30 μM TP solutions, and freeze-dried TP-PLGA-Au@RGD/HA hydrogels solutions (equivalent TP of 13 μM TP solutions), respectively. And TP-PLGA-Au@RGD/HA hydrogels groups underwent 10 min NIR irradiation treatment. The results showed that the cell anti-proliferation effect on 13 μM TP solutions group was significantly less relative to the effect on the 30 μM TP solutions group owing to the lower TP concentration in the 13 μM TP solutions group. The results showed that nearly-similar cell proliferation inhibition rates in the 30 μM TP solutions group and TP-PLGA-Au@RGD/HA hydrogels solutions combined with the NIR group. However, the 30 μM TP solutions group was almost double of TP concentration used in free conditions. These results indicated that TP-PLGA-Au@RGD/HA hydrogels exhibited a synergistic effect with NIR irradiation in inhibiting cell proliferation and had higher efficiency.

### 3.4 Anti-Proliferative Effect of Hydrogel-Treated RA-FLS Is Mediated Through the mTOR/p70S6K Signaling Pathway

Distinctive features of RA are synovial inflammation and synovial cell hyperplasia (Tang et al., 2020). RA-FLS are the key components of invasive synovium and play an important role in the initiation and progression of destructive joint inflammation (Pantuck et al., 2007). Chemokines (such as CXCL1, CXCL5, and G-CSF), inflammatory cytokines (such as TNF-a, IL-1b, and IL-6), and inflammatory mediators (such as TLR-2, TLR-4, iNOS) released by RA-FLS can promote infiltration of DCs, monocytes, neutrophils, macrophages, B cells, and T cells into joints. Infiltration of these immune cells induces chronic inflammation and joint destruction (Tang et al., 2020). Recent studies reported that the mTOR/p70S6K signaling pathway played a major role in regulating cell survival and apoptosis, and was upregulated in RA-FLS (Huang et al., 2020). mTOR is upregulated in a variety of cancers and is implicated in the regulation of cancer cell invasion. In addition, the expression mTOR is associated with poor prognosis in cancer (An et al., 2010; Laragione and Gulko, 2010; Meng et al., 2006; Pantuck et al., 2007; F.; Zhou et al., 2005; L.; Zhou et al., 2010). The p70S6K1 regulates cell growth by inducing protein synthesis components (F. Zhou et al., 2005). Therefore, analyses were conducted to explore whether the anti-proliferative effect of hydrogels on RA-FLS was mediated through inhibition of the mTOR/p70S6K signaling pathway. Therefore, protein expressions levels of total mTOR, p70S6K, p-mTOR, p-p70S6K in each group were explored by western blotting analysis (Figure 6B). The results showed significant decrease in the levels of phosphorylated mTOR in RA-FLS treated with hydrogels combined with 10 min NIR irradiation at 0.38 W/cm^2^ (Figure 6B, p-mTOR/total median reduction rate at 54%). Moreover, administration of hydrogels combined with 10 min NIR irradiation decreased the level of the phosphorylated form of the mTOR substrate, p70S6K in RA-FLS (Figure 6B, p-p70S6K/total median reduction rate of 38%). These results indicated that hydrogels inhibited the mTOR/p70S6K signaling pathway in RA-FLS thus inhibiting the proliferation of these cells.

### 3.5 *In Vivo* Targeted Effects and NIR Imaging

**FIGURE 7:**
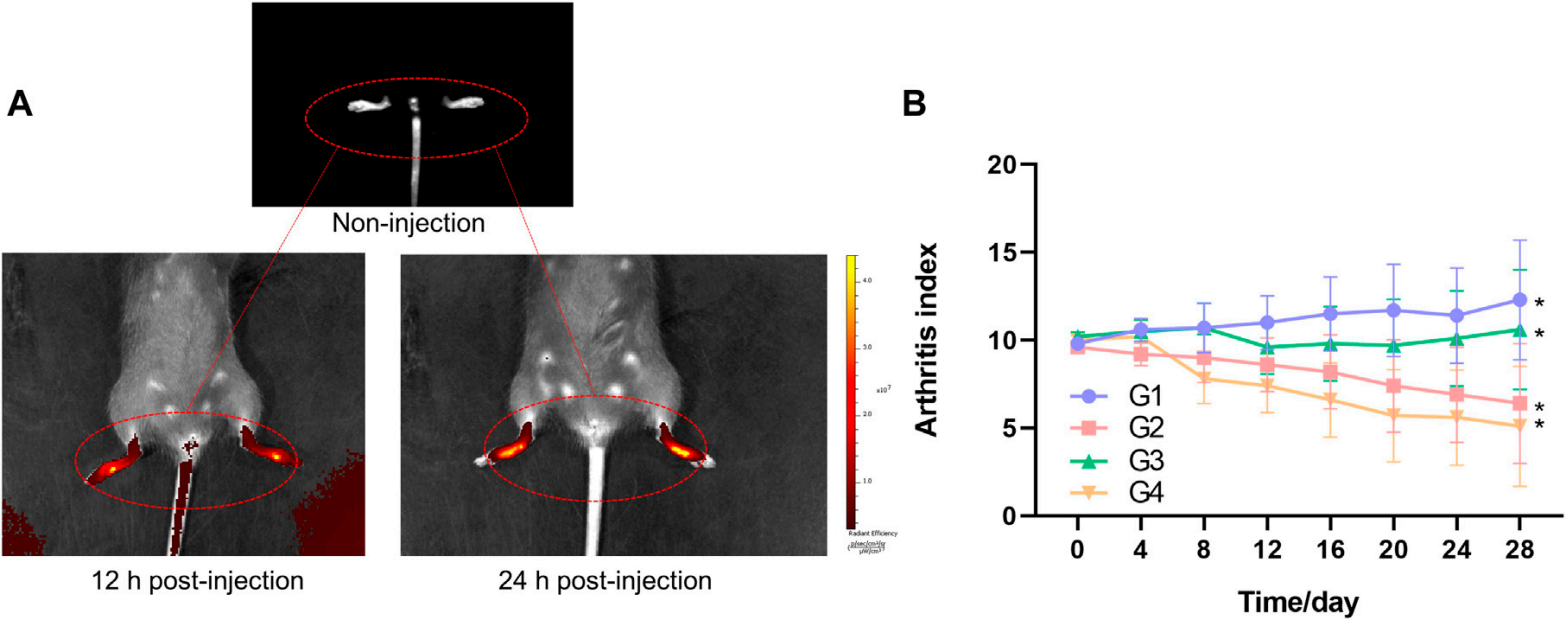
**(A)** Fluorescence images of inflamed paws of CIA mice of non-injection, 12 h post-injection, and 24 h post-injection of TP-PLGA-Au@RGD-Cy7/HA hydrogels, and mice were intraarticularly administered. **(B)** Arthritis index versus time for CIA mice. Arthritis indices were significantly different among groups (*p < 0.05).

CIA mice model was established to explore the targeted effects on inflamed joints *in vivo* through fluorescence imaging of TP-PLGA-Au@RGD-Cy7/HA hydrogels. Mice were intraarticularly administered with TP-PLGA-Au@RGD-Cy7/HA hydrogels solution and Cy7. Hydrogels endowed the composite systems with unique optical properties owing to the superior capabilities of AuNPs (Shin et al., 2013; Ye and Loh 2013). Injected hybrid hydrogels were monitored by *in vivo* NIR absorbance at 12 and 24 h (Figure 7A). The findings showed that the color of inflamed paws changed with time, indicating that nanoparticles were selectively delivered to the inflamed areas and accumulated at the inflamed joint. Notably, the color area of inflamed paws of CIA mice changed over time due to the localization of TP-PLGA-Au@RGD NPs in the inflamed paws (Figure 7A). These results indicated that TP-PLGA-Au@RGD NPs were preferentially delivered to the inflamed region through active targeting, and effectively accumulated on the inflamed paws.

### 3.6 *In Vivo* Efficacy of Prepared Hydrogels

During the design process of the experiment, we selected 33 male DBA/1J mice. After 1 week of adaptive feeding, 6 mice were randomly selected as the healthy group and the remaining 27 mice were used to establish the CIA mice model. Only 24 mice were successfully modeled (arthritis index ≥2). The successfully modeled mice were divided into 4 groups by random number method, each with 6 mice, namely G1, G2, G3, and G4, to explore the treatment efficacy of hydrogels. TP-PLGA-Au@RGD/HA hydrogels were prepared by mixing the TA-HA solution, TP-PLGA-Au@RGD NPs, H_2_O_2_ solution, and HRP solution, and were injected *in situ* immediately. H_2_O_2_ is an oxidant of HRP and reduced to water, and HRP as a model catalyst, which induces the oxidative coupling of the phenol moiety. The gelation time was recorded when the hydrogel formed and stopped flowing upon vial tilting. This mixture began to form hydrogel immediately *in vitro* and was completely solidified after 3 minutes, suggesting that the hydrogel formation time would be quick enough for *in vivo* applications. The final concentrations of H_2_O_2_ and HRP were 529 μ mol/L and 0.2 U/ml in the TP-PLGA-Au@RGD/HA hydrogels precursor solution, respectively.

Arthritis indices of the four groups over time were presented in Figure 7B. Treatment groups showed a decrease in arthritis indices with some variations depending on the day compared to the mice administered with G1. The decrease in arthritis indices in G3 was slow until about day 20 and then showed a significant increase. G3 comprised mice treated with TP-PLGA-Au@RGD/HA hydrogels with non-NIR irradiation, and arthritis indices in this group were lower relative to the arthritis indices in G1. Arthritis indices of G4 were lower compared with the levels in G2. This difference between G4 and G2 can be attributed to the photothermally controlled drug release in G4. Release of more than 10% of the TP occurred within 12 h when the inflamed paws were exposed to NIR light (G4), which comprised a significantly lower TP dosage compared to the dose administered in G2, however, a high therapeutic effect was observed in G4. These results were consistent with the pattern of that observed for cell proliferation inhibition, indicating that the TP-PLGA-Au@RGD/HA hydrogels system was beneficial for controlled drug delivery and with much higher efficiency.

### 3.7 Histopathological Analysis

**FIGURE 8:**
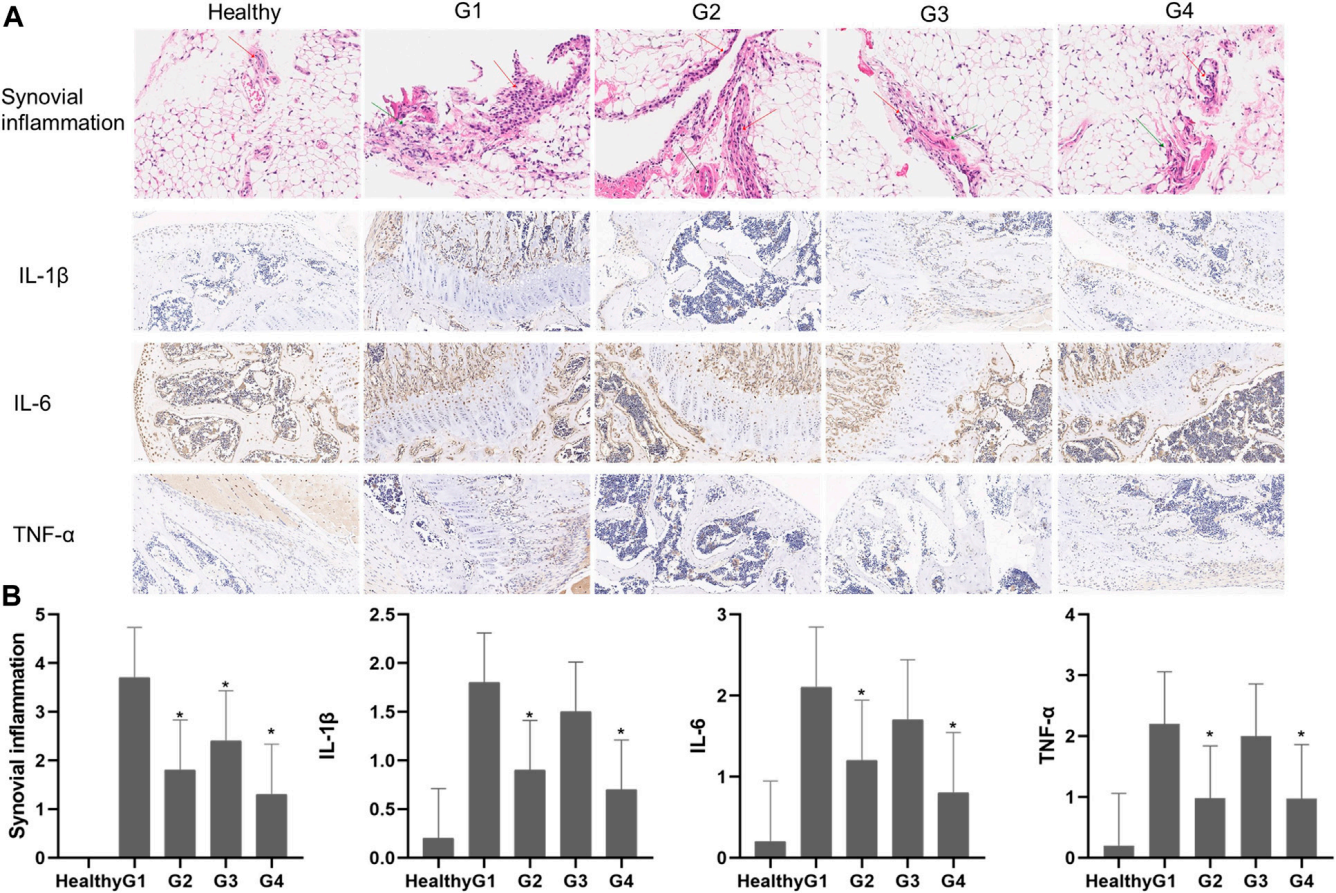
**(A)** Histological findings of synovial tissues from healthy mice and CIA mice on day 28 after different treatments. H&E (synovial inflammation, original magnifications × 100, and immunohistochemical staining for IL-1β, IL-6, and TNF-α, original magnifications × 20) **(B)** Semiquantitative analysis of histopathological evaluation (synovial inflammation and immunohistochemical staining for IL-1β, IL-6, and TNF-α). Asterisks (*) represent significance compared with the untreated mice at *p < 0.05.

Histological analysis of joint tissues was performed on day 28 after intraarticular administration of the different treatments to further explore the effects of targeted photothermal-chemo treatment (Figure 8A). Histological analysis showed no significant abnormality in the tissue structure of the healthy group. The layer between fat cells and collagen fibers was clearly demarcated, and collagen fibers were neatly arranged. A single-layer cubic epithelial structure and low levels of inflammatory cell infiltration were observed. Severe infiltration of inflammatory cells was observed in the joint sections of untreated mice (G1). The layer between fat cells and collagen fibers was unclear in tissues from the G1 group. In addition, collagen fibers were disorderly arranged, and a single-layer cubic epithelial structure and a high number of inflammatory cell infiltration were observed. Infiltration of inflammatory cells was significantly reduced in mice treated with TP solution four times a week (G2) and mice treated with TP-PLGA-Au@RGD/HA hydrogels combined with NIR irradiation (G4). The tissue structure in G2 was slightly affected, collagen fiber arrangement was disordered and a single-layer cubic epithelial structure was observed. A low level of inflammatory cell infiltration was observed in the tissue. The layer between the fat cell and the collagen fibers was clearly demarcated in tissues in G4, and the collagen fibers were neatly arranged. A single-layer cubic epithelial structure and a small amount of inflammatory cell infiltration were observed in the tissue. The tissue structure in G3 was moderately affected, the layer between fat cells and the collagen fibers was unclear, and collagen fibers were disorderly arranged. In a monolayer cubic epithelial structure a relative amount of inflammatory cell infiltration was observed in the tissue. Synovitis inflammation scores in G2 and G4 groups were significantly lower (*p < 0.05*) relative to the scores in the G1 group, and G4 was lower than G2 (Figure 8B).

Previous studies reported that pro-inflammatory cytokines such as TNF-α, IL-β, and IL-6 play important roles in inflammatory response (Kuncirova et al., 2014) and were implicated in the pathogenesis of RA. Immunohistochemical staining showed that the levels of TNF-α, IL-6, and IL-1β were increased around the joint in the G1 group, whereas the expression levels were significantly decreased in joint tissues in G2 and G4 groups (Figures 8A,B). The results showed no significant differences between expression levels of pro-inflammatory cytokines in G2 and G4, however, G4 is still lower than G2. These results, together with the *in vitro* results, demonstrated that TP-PLGA-Au@RGD/HA hydrogels combined with NIR irradiation had significantly higher effects with a low dosage of TP.

### 3.8 CT Imaging Analysis

**FIGURE 9:**
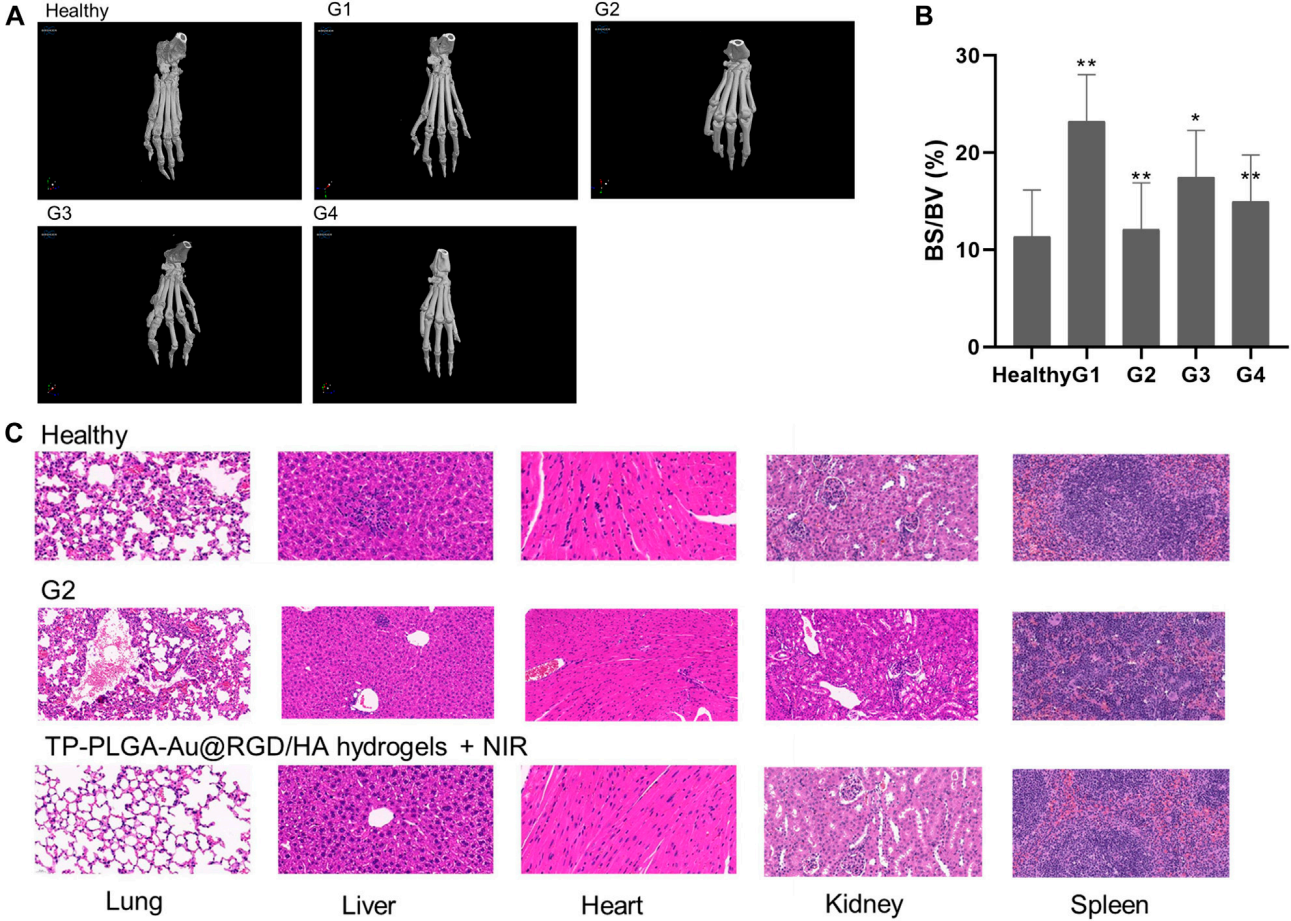
**(A)** Micro-computed tomography images of hind paws of CIA mice in different treatment groups. **(B)** Effects of G4 on bone destruction in CIA mice. Bone volume/total volume (BV/TV; %) in CIA mice. *p < 0.05 versus G1; **p < 0.01 versus G1. **(C)** Histological sections of major organs extracted on day 28 after administration of health (top), TP-PLGA-Au@RGD/HA hydrogels combined with NIR irradiation (bottom, G4) and G2. Images were acquired under ×20 magnification.

Three-dimensional micro-CT imaging was used to explore changes in the bones of paws of CIA mice treated with hydrogels (Figure 9A). Bone parameters (bone volume/total volume; BV/TV) were explored (Figure 9B). Severe bone destruction of paws was observed in CIA mice administered with saline (G1). Bone structures of mice in G2 and G4 groups were relatively well preserved compared with the bone structures of mice in G1. The bone volume of paws of CIA mice was determined to explore the level of bone preservation. The bone volume of the paws of mice treated with TP-PLGA-Au@RGD/HA hydrogels and NIR irradiation showed significant preservation. These results indicated the nearly-similar therapeutic effect of TP-PLGA-Au@RGD/HA hydrogels with NIR irradiation and G2, however, G4 was a lower TP concentration.

### 3.9 *In Vivo* Toxicity of Hydrogels

Histological analysis of major organs (spleen, liver, lung, heart, and kidney) was conducted on day 28 after administration of different treatments to explore the toxicity of TP-PLGA-Au@RGD/HA hydrogels combined with NIR (G4), G2 and healthy group (Figure 9C). The lung tissue of G4 was a little different from the healthy group; however, the damage was mild. And the major organs of the mice in the G2 were slightly tissue damaged compared to healthy mice. The findings showed that there was no significant tissue damage in G4 mice compared with the healthy group, indicating that TP-PLGA-Au@RGD NPs did not accumulate in major organs and did not induce significant *in vivo* toxicity.

## 4 Conclusion

In the present study, novel TP-PLGA-Au@RGD/HA hydrogels were successfully prepared as a drug delivery system with anti-inflammation, chondroprotection, photothermal-chemo therapy, and *in vivo* imaging properties for the treatment of RA. *In vivo,* NIR absorbance images showed that the nanoparticles selectively accumulated in the inflamed regions of CIA mice after administration of the hydrogels into joints of CIA mice. NIR irradiation increased the temperature of the exposed area and accelerated the TP release rate from the TP-PLGA-Au@RGD/HA hydrogels, inducing photothermal-chemo therapy. Treatment with hydrogels combined with NIR irradiation had significantly higher effects with a smaller dosage of TP and low toxicity compared to conventional single treatment with TP. Furthermore, the findings indicated that the TP-PLGA-Au@RGD/HA hydrogels inhibited inflammation by partly suppressing invasion and migration of RA-FLS, and by blocking phosphorylation factors in the mTOR/p70S6K pathway. These results implied that targeted photothermal-chemo therapy using hydrogels was a useful and effective strategy for maximizing therapeutic efficacy and minimizing dose-related side effects during the treatment of RA.

## Data Availability

The original contributions presented in the study are included in the article/Supplementary Material, further inquiries can be directed to the corresponding authors.
