# Supplementary material for: Hyaluronic Acid Hydrogels Hybridized With Au-Triptolide Nanoparticles for Intraarticular Targeted Multi-Therapy of Rheumatoid Arthritis
Source: Front Pharmacol. 2022 May 27;13:849101. doi: 10.3389/fphar.2022.849101 (PMC9197263; doi:10.3389/fphar.2022.849101)
Supplement: Supplementary file 1 [file DataSheet1.docx]

**Supplementary materials**

**Hyaluronic acid Hydrogels Hybridized with Au-Triptolide Nanoparticle for Intra-articular targeted multi-therapy of Rheumatoid** **Arthritis**

Chenxi Li^1^, Rui Liu^1^, Yurong Song^1^, Youwen Chen^1^, Dongjie Zhu^1^, Liuchunyang Yu^1^, Qingcai Huang^1^, Zhengjia Zhang^1^, Zeyu Xue^1^, Zhenglai Hua^1^, Cheng Lu^2*^, Aiping Lu^3*^, Yuanyan Liu^1*^

**Affiliation**

^1^ School of Chinese Materia Medica, Beijing University of Chinese Medicine, Beijing 100102, China

^2^ Institute of Basic Research in Clinical Medicine, China Academy of Chinese Medical Sciences, Beijing 100700, China

^3^ School of Chinese Medicine, Hong Kong Baptist University, Kowloon, Hongkong, China

**Contact Information for Corresponding Author：**

Dr. Yuanyan Liu, School of Chinese Materia Medica, Beijing University of Chinese Medicine, Beijing 100029, China. Tel: +86 10 84738658, Fax: +86 10 84738611. E-mail address: [yyliu_1980@163.com](mailto:yyliu_1980@163.com).

Dr. Cheng Lu, Institute of Basic Research in Clinical Medicine, China Academy of Chinese Medical Sciences, Beijing 100700, China. E-mail address: [lv_cheng0816@163.com](mailto:lv_cheng0816@163.com).

Dr. Aiping Lu, School of Chinese Medicine, Hong Kong Baptist University, Kowloon, Hongkong, China. E-mail address: lap64067611@126.com.

Part of the data on this manuscript has been preprinted [DOI: 10.21203/rs.3.rs-750774/v1].

**
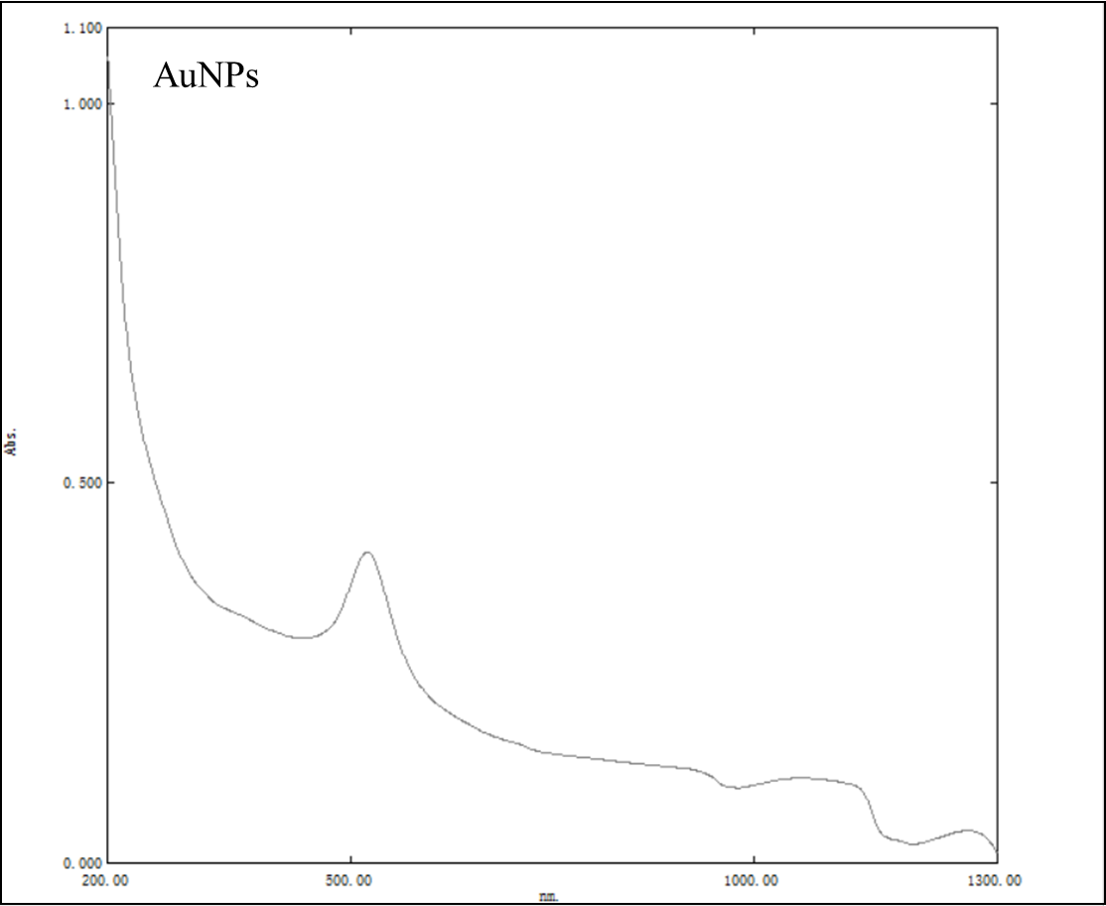
**

**Fig.S1 UV-vis-NIR absorbance spectra of AuNPs.**

**(a)**

**
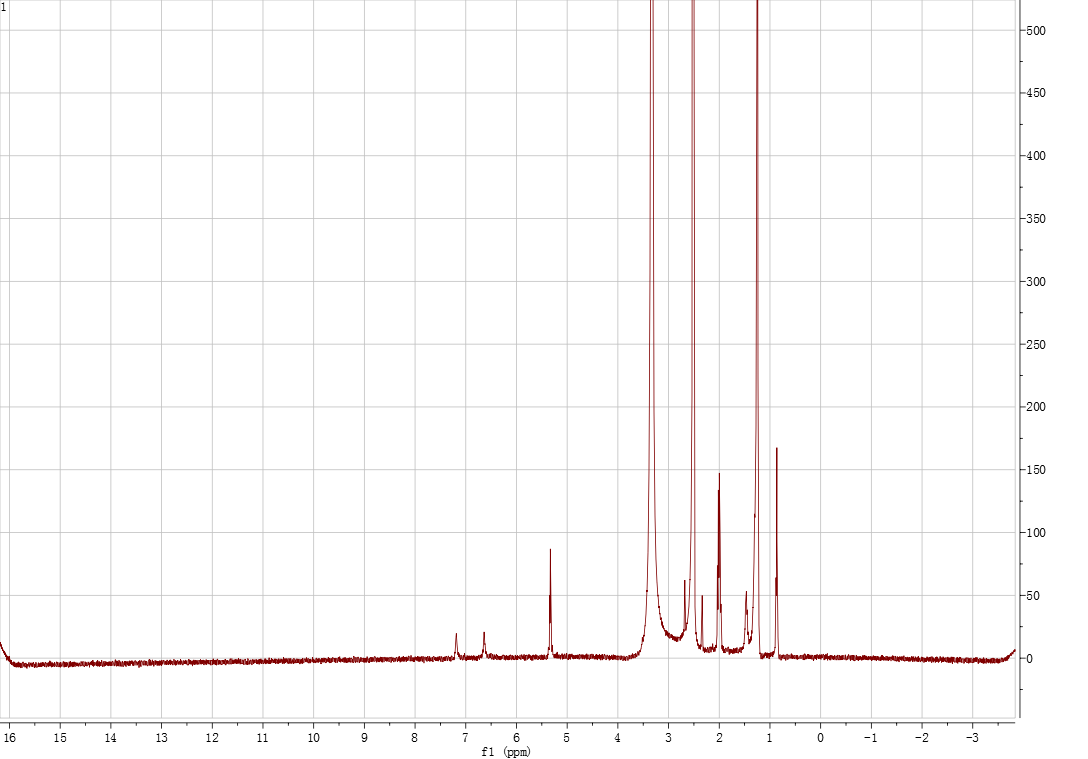
**

**(b)**

**
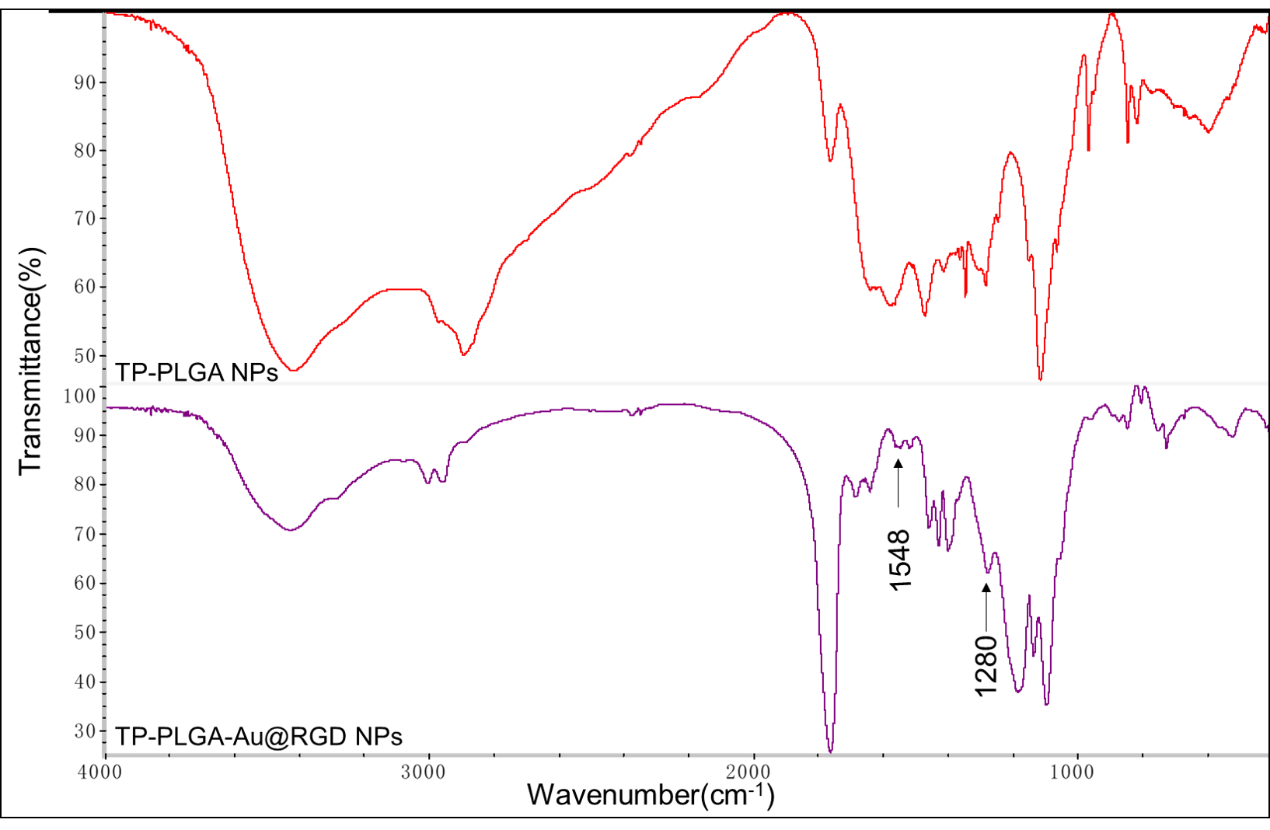
**

**Fig.S2 (a) ^1^H NMR spectra of modified HA. (b) FT-IR spectra of the TP-PLGA NPs and TP-PLGA-Au@RGD NPs**

**
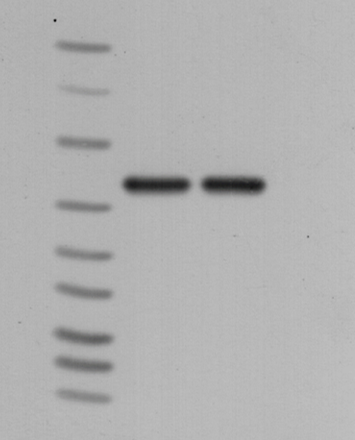
**

**p70s6k**

**
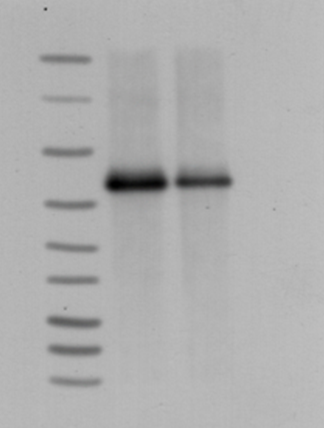
**

**p-p70s6k**

**
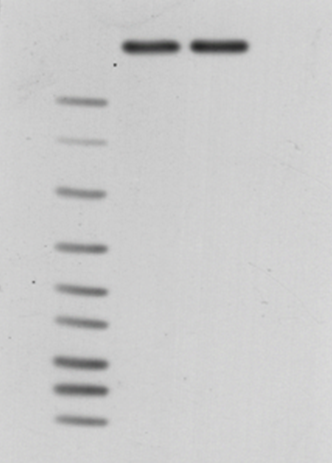
**

**mTOR**

**
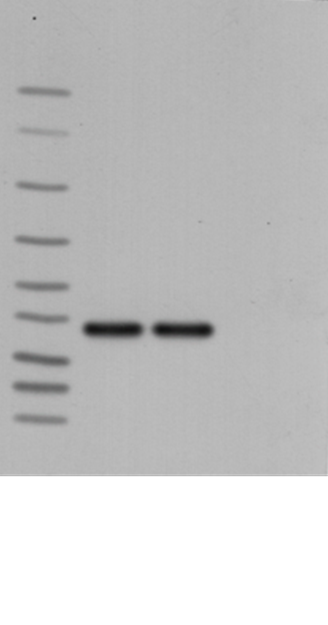
**

**β-actin**

**
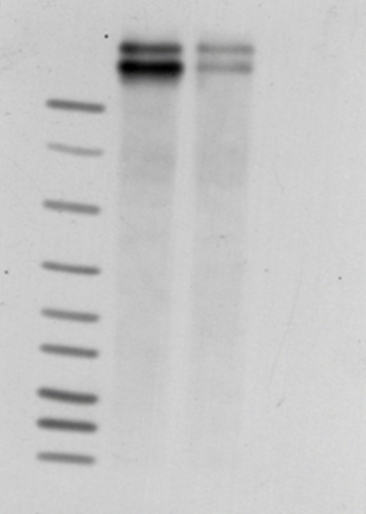
**

**p-mTOR**

**Fig.S3 The images of Western Blot. The left side represents the maker, the middle represents the control group and the right side represents the TP-PLGA-Au@RGD/HA hydrogel group.**
